# Comment on Kass et al. The Development of the Radial Bow in Children: Normative Values. *Children* 2025, *12*, 101

**DOI:** 10.3390/children13081100

**Published:** 2026-08-18

**Authors:** Barış Kafa, Berat Yüksel

**Affiliations:** Department of Orthopaedics and Traumatology, Faculty of Medicine, Hacettepe University, Ankara 06230, Turkey; beratyuksel@hacettepe.edu.tr

We read with great interest the article by Kass et al. reporting normative values of the radial bow in children [1]. While establishing pediatric normative data is of significant value to the literature, we wish to address a critical methodological discrepancy that may affect the validity and reproducibility of these values.

The authors define “depth at maximal radial bow” as the distance from the radial cortex to the ulnar cortex [1]. This approach fundamentally deviates from the originally described and most widely cited method established by Schemitsch and Richards, which defines depth as the perpendicular distance from a fixed longitudinal, intra-radial reference line to the radial cortex [2] (Figure 1). This is also the method that was used to establish the pediatric normative values of Firl and Wünsch [3], with which the authors compare their own results [1]; a direct numerical comparison between the two datasets is therefore not methodologically equivalent.

We believe that using the ulnar cortex as a reference landmark instead of an independent reference line introduces significant confounding factors:

1. Dependency on Ulnar Morphology: The ulna is not a uniformly straight bone, and its contour varies considerably between individuals; in a radiographic series of 175 children, the dorsal border of the ulna was concave in 15.4%, straight in only 51.4% and convex in 33.2% of cases [4]. By measuring the distance between the two bones, the authors are reporting a summation of both radial and ulnar curvatures rather than the isolated, intrinsic morphology of the radius.
2. Inappropriate Reference Landmark: Both forearm bones undergo continuous, age-dependent change throughout skeletal maturation [5], and the radial bow itself has been shown to vary with age on three-dimensional imaging [6]. Defining the bow of one growing bone by its distance to a second, independently growing bone therefore superimposes two developmental trajectories. In morphometric terms, using one changing variable (the radioulnar distance) to define another (the radial bow) creates a “moving target” error that compromises the accuracy of the established normative data.
3. Sensitivity to Projection and Forearm Rotation: Any measurement that depends on the position of one forearm bone relative to the other is inherently sensitive to limb positioning and radiographic projection. Only 12.6% of routinely obtained pediatric forearm radiographs have been shown to represent true lateral projections, and malpositioning of the limb significantly altered the radiographic assessment of ulnar bowing [4]. The Schemitsch and Richards method, which relies exclusively on intra-radial landmarks, is inherently more resilient to such variation.

In conclusion, we believe that for normative values to be accurate and comparable with the existing literature, the radial bow must be measured as an intrinsic property of the radius, independent of the adjacent ulnar anatomy.

## Figures and Tables

**Figure 1:**
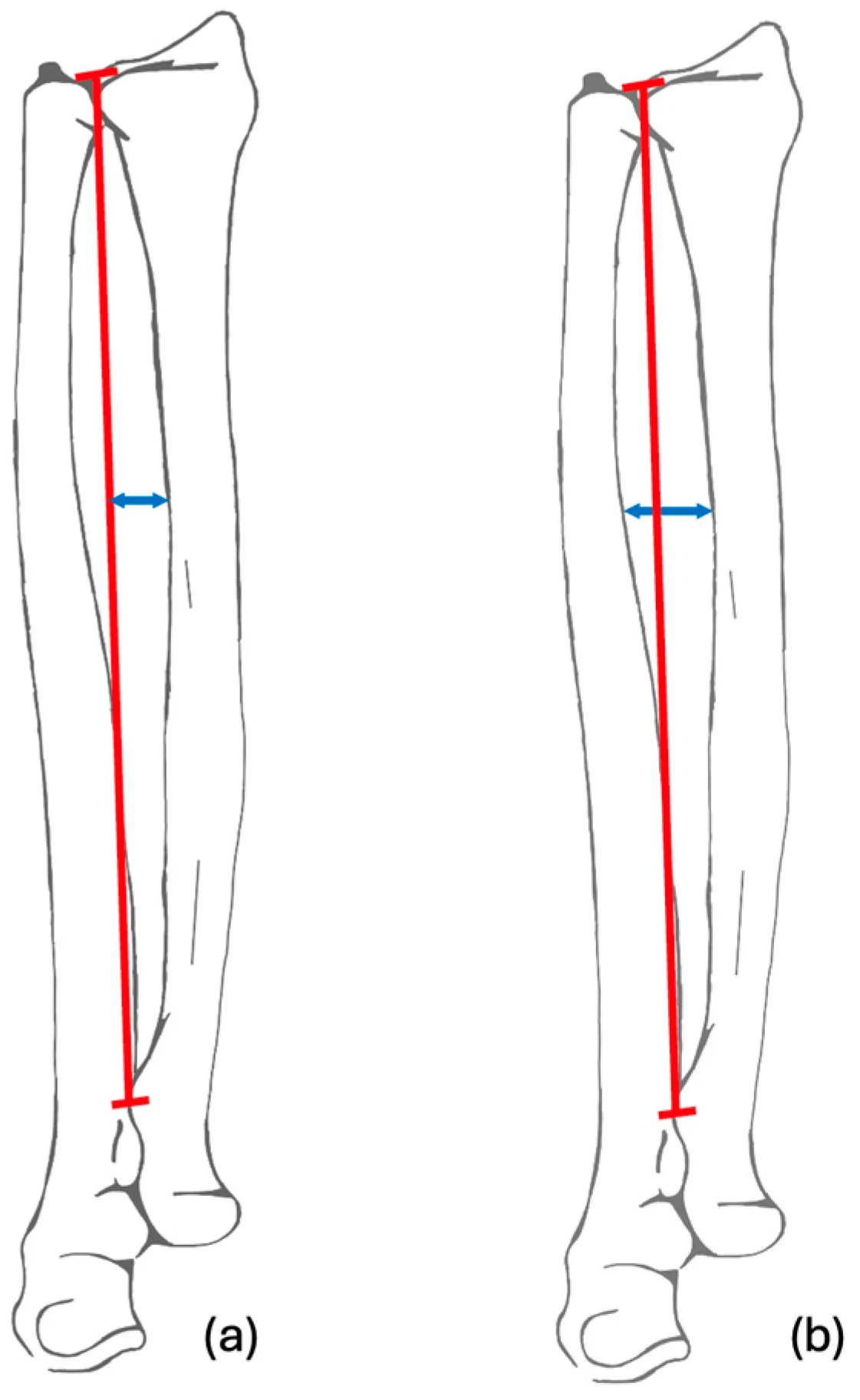
Schematic presentation contrasting the two methodologies for measuring maximal radial bow depth. (**a**) The traditional Schemitsch and Richards technique: Depth is measured as the perpendicular distance from an independent, intra-radial longitudinal reference line (red line) to the apex of the radial cortex. This isolates pure radial morphology. (**b**) The technique used by Kass et al.: Depth is measured as a composite inter-cortical distance directly from the radial cortex to the ulnar cortex. This alternative approach inherently incorporates confounding variables such as the patient’s intrinsic ulnar curvature and age-dependent interosseous distance.

## Data Availability

No new data were created or analyzed in this study. Data sharing is not applicable to this article.
